# Impact of preprocedural atrial fibrillation on immediate and long-term outcomes after successful percutaneous mitral valvuloplasty of significant mitral stenosis

**DOI:** 10.1007/s12928-016-0434-9

**Published:** 2016-10-05

**Authors:** Shiro Miura, Takeshi Arita, Takenori Domei, Kyohei Yamaji, Yoshimitsu Soga, Makoto Hyodo, Shinichi Shirai, Kenji Ando

**Affiliations:** 1 Department of Social, Human and Mathematical Sciences, University of Southampton, Highfield, Southampton, Hampshire SO17 1BJ UK; 2 Department of Medicine and Biosystemic Science, Kyushu University Graduate School of Medicine, Fukuoka, Japan; 3 Department of Cardiology, Kokura Memorial Hospital, Kitakyushu, Japan

**Keywords:** Mitral stenosis, Percutaneous mitral valvuloplasty, Atrial fibrillation, Long-term outcome

## Abstract

Optimal time to perform percutaneous mitral valvuloplasty (PMV) for patients with significant mitral stenosis (MS) and atrial fibrillation (AF) remains controversial. We sought to identify prognostic factors and evaluate long-term clinical outcomes after PMV of 77 consecutive patients with MS with a mitral valve area (MVA) <1.5 cm^2^. According to baseline heart rhythm, these patients were divided into sinus rhythm (SR; *n* = 24) and AF (*n* = 53) groups. The study endpoint was defined as a composite of all-cause mortality, admission for heart failure, mitral valve surgery, repeated PMV, and major cerebral vascular accident during follow-up. After successful PMV, there was no significant difference between the two groups in post-MVA and post-mitral mean pressure gradient. However, the New York Heart Association Functional Classification post-procedure was worse in the AF group (*p* < 0.01). In the AF group, event-free survival during follow-up was significantly lower compared with that of the SR group (*p* = 0.016). Independent predictors of clinical events were AF [hazard ratio (HR), 2.73; 95 % confidence interval (CI), 1.04–9.36; *p* = 0.03] and pulmonary artery systolic pressure (HR 2.57; 95 % CI 1.18–5.47; *p* = 0.017). Patients with AF at baseline were significantly associated with worse symptoms and higher event rates after successful PMV compared with those with SR. The clinical benefit of PMV may be considered for patients with MVA <1.5 cm^2^ before the onset of AF.

## Introduction

Since its introduction in 1984 by Inoue [1], percutaneous mitral valvuloplasty (PMV), which is also well known as percutaneous transluminal mitral commissurotomy (PTMC), has been established as a safe and effective procedure for the treatment of symptomatic mitral stenosis (MS) [2–4]. The decrease in the incidence of acute rheumatic fever in developed countries led to a sharp decrease in the incidence of MS [5]. This decrease modifies clinical presentation, and MS is now encountered in patients with the characteristics as follows: advanced age, atrial fibrillation (AF), history of surgical commissurotomy, repeated PMV, and severe impairment of valve anatomy. Previous studies have demonstrated that valve morphology predicts event-free survival [6, 7]. Therefore, mitral valve replacement (MVR) is the ideal therapeutic option for patients with remarkable mitral valve degeneration.

The development of AF is a common condition and an essential sequela in patients with MS that is associated with hemodynamic and clinical deterioration. AF may affect symptoms and clinical outcomes in patients with MS and AF. For example, AF is associated with suboptimal immediate results and midterm outcome after PMV [8–10]. However, it remains controversial whether AF is an essential independent predictor of long-term outcomes after successful PMV for predominantly pliable MS. Therefore, we sought to identify prognostic factors and evaluate long-term clinical outcomes after PMV in patients with MS with sinus rhythm (SR) and AF.

## Methods

### Study population

**Fig. 1:**
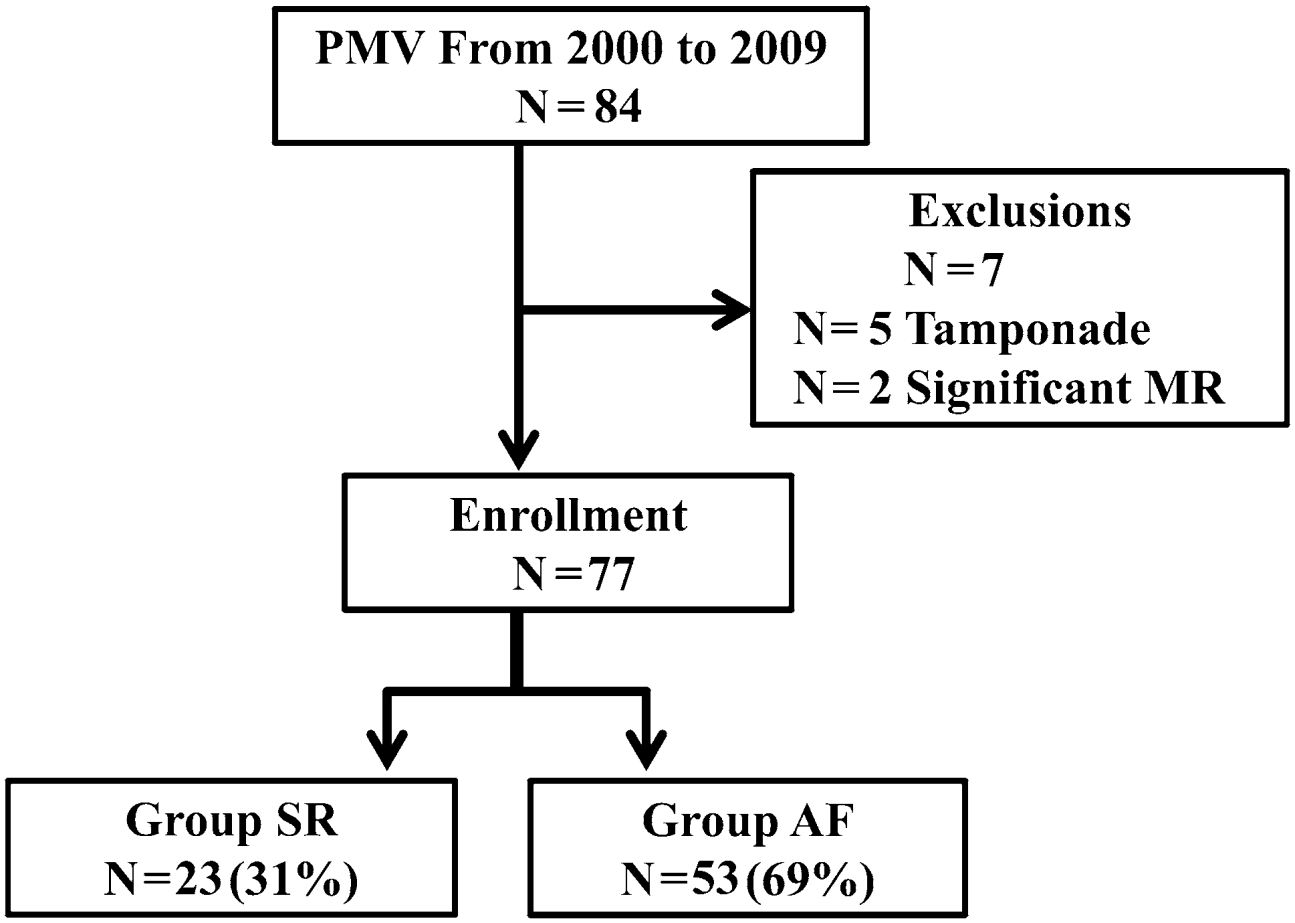
Description of the patient population. *MS* mitral stenosis, *MR* mitral regurgitation, *PMV* percutaneous mitral valvuloplasty, *AF* atrial fibrillation, *SR* sinus rhythm

We retrospectively enrolled 84 consecutive patients with MS who underwent PMV in our institution between March 2000 and March 2009. Following 7 patients were excluded: 5 patients with cardiac tamponade and 2 patients with severe mitral regurgitation (MR). Finally, 77 patients who achieved immediate procedural success (defined as mitral valve area (MVA) ≥1.5 cm^2^ and MR ≤¾) were analyzed. Patients were divided according to baseline heart rhythm into a SR group (*n* = 24) and AF group (*n* = 53) (Fig. 1). After PMV, they were effectively treated with warfarin. Further, those who converted from SR into AF during clinical follow-up after PMV were also administered warfarin. This study was approved by our Institutional Review Board.

### Percutaneous mitral valvuloplasty

PMV was performed using the Inoue balloon technique [11]. Briefly, the procedure was performed by experienced interventional cardiologists, and conventional hemodynamic parameters were monitored during the procedure. Initial balloon size was selected according to body surface area and mitral annular size measured using echocardiography. Left ventriculography was performed before and after the last balloon inflation. Written informed consent was obtained from all patients prior to PMV.

### Echocardiographic evaluation

All patients underwent comprehensive two-dimensional and color Doppler echocardiographic evaluations on the day before and 24 h after undergoing PMV. The morphological features of the mitral valve were assessed, and the total echocardiographic score (Wilkins scores) was calculated by adding the scores for leaflet mobility, thickness, calcification, and subvalvular lesions [12]. MVA was calculated from the Doppler study using the pressure half-time method and was measured using direct planimetry at the parasternal short-axis view. The mean pressure gradient (MPG) across the mitral valve was assessed using the continuous-wave Doppler method. In patients with AF, measurements were repeated 5 times during different cardiac cycles, and the mean value was used for data analysis. Pulmonary artery systolic pressure (PASP) was estimated as the sum of right atrial pressure and the pressure gradient between the right ventricle and right atrium during systole, which was calculated using the modified Bernoulli equation. As per the recommendations of the American Society of Echocardiography [13], right atrial pressure was estimated according to the echocardiographic characteristics of the inferior cava. After PMV, the extent of the commissurotomy or separation of the commissures, primarily at the parasternal short-axis and the apical two-chamber views, were assessed along with the development of MR. Color Doppler flow imaging was performed with multiple orthogonal parasternal and apical views for diagnosis and quantification of MR after PMV. The severities of MR and tricuspid regurgitation were judged according to the American College of Cardiology/American Heart Association guidelines [14].

### Study endpoint and definition of clinical events

Clinical follow-up data were obtained from interviews with patients, relatives, or attending physicians. The study endpoint was the cumulative incidence of major cardiovascular adverse events, defined as a composite of all-cause mortality, hospitalization required for heart failure (HF), repeated PMV, MVR, and major cerebral vascular accident (CVA) that included ischemic stroke and hemorrhagic stroke.

### Statistical analysis

Continuous data are presented as mean ± standard deviation (SD). Categorical variables were compared between groups using the Chi-square test or Fisher’s exact test, as appropriate. Continuous variables were compared between groups using the Student unpaired *t* test or the Mann–Whitney test, according to the distribution. The Kruskal–Wallis test was used to compare more than two groups. Kaplan–Meier analysis was used to determine the event-free survival rate, and log-rank test was used to analyze differences in survival rates between the SR and AF groups. A two-sided probability (*p*) value <0.05 was considered statistically significant.

The effect of significant clinical factors on clinical events was assessed using univariate and multivariate Cox proportional hazards models. In the predictive analysis, continuous variables were categorized into two subgroups according to median values. A Cox multivariate analysis including all variables with *p* values <0.1 derived from the Cox univariate analysis, and proportional assumptions that were generally fair, were used to determine the predictive factors of clinical events. All statistical analyses were performed using JMP, version 8.0.2 (SAS Institute Inc, Cary, NC).

## Results

### Patient characteristics and immediate results

**Fig. 2:**
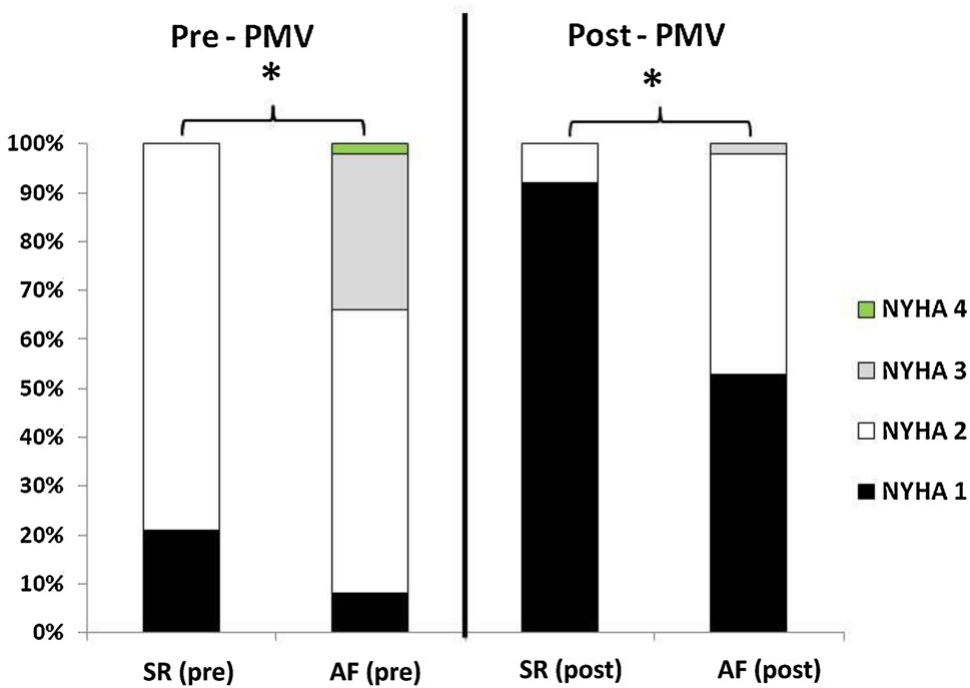
Functional capacity according to NYHA classes before and after PMV. *NYHA class* New York Heart Association functional class, *PMV* percutaneous mitral valvuloplasty, *AF* atrial fibrillation, *SR* sinus rhythm *p < 0.01

Patients’ baseline characteristics and initial results are shown in Table 1. The average Inoue balloon diameter was 24.6 ± 1.8 mm. The two groups had similar baseline characteristics, including age, sex, risk factors for atherosclerosis, and histories of mitral valve intervention and hospitalization because of HF. Previous major embolic events were significantly higher in the AF group. The frequencies of New York Association functional classes (NYHA class) III and IV at baseline were significantly higher in the AF group compared with those of the SR group (34 vs. 0 %, *p* < 0.01). The immediate hemodynamic and echocardiographic changes are shown in Table 2. All patients who underwent PMV had an echocardiographically suitable mitral valve for intervention (echocardiographic score ≤9). The only significant differences between the groups were observed in left atrium size and echocardiographic score. There was no significant difference between the two groups in post-MVA and post-mean mitral MPG, whereas NYHA class post-procedure was worse in the AF group (*p* < 0.01) (Fig. 2).

**Table 1:** Comparison of patients’ baseline clinical characteristics

	Total (*n* = 77)	SR (*n* = 24)	AF (*n* = 53)	*p* value
Age (years)	62 ± 9	61 ± 9	62 ± 9	0.64
Sex (female)	54 (71)	19	35	0.25
NYHA class				
I–II	59 (77)	24	35	<0.01
III–IV	18 (23)	0	18	<0.01
Previous PMV	9 (12)	4	5	0.29
Previous OMC	12 (16)	1	11	0.63
Previous CVA	11 (14)	0	11	0.02
HTN	13 (17)	2	11	0.18
DM	14 (18)	3	11	0.43
CKD	9 (12)	2	7	0.58
History of HF admission	11 (14)	3	8	0.77

Values represent the mean ± SD or *n* (%)

*SR* sinus rhythm, *AF* atrial fibrillation, *NYHA class* New York Heart Association Functional Class, *PMV* percutaneous mitral valvuloplasty, *OMC* open mitral commissurotomy, *CVA* cerebral vascular accident, *HTN* hypertension, *DM* diabetes mellitus, *CKD* chronic kidney disease, *HF* heart failure

**Table 2:** Comparison of echocardiographic characteristics and immediate results

	Total (*n* = 77)	SR (*n* = 24)	AF (*n* = 53)	*p* value
LVEF (%)	61 ± 13	62 ± 12	61 ± 14	0.63
LVDD (mm)	47 ± 6	46 ± 6	48 ± 6	0.22
LAD (mm)	53 ± 8	48 ± 6	55 ± 8	<0.001
MVA (cm^2^)				
Pre-procedure	1.1 ± 0.2	1.1 ± 0.2	1.1 ± 0.2	0.70
Post-procedure	1.6 ± 0.3	1.6 ± 0.3	1.6 ± 0.2	0.84
Mitral MPG (mmHg)				
Pre-procedure	9.0 ± 4.4	8.8 ± 5.8	9.2 ± 4.0	0.78
Post-procedure	5.3 ± 2.6	3.9 ± 1.7	5.7 ± 2.8	0.13
Echocardiographic score	6.6 ± 1.0	6.0 ± 1.4	6.8 ± 0.8	0.01
Baseline MR (III or IV)	1 (1)	0	1	0.68
Baseline TR (III or IV)	8 (10)	2	6	0.99
Baseline PASP (mmHg)	41 ± 10	41 ± 15	41 ± 8	0.84

Values represent the mean ± SD or *n* (%)

*SR* sinus rhythm, *AF* atrial fibrillation, *LVEF* left ventricular ejection fraction, *LVDD* left ventricular diastolic dimension, *LAD* left atrial dimension, *MVA* mitral valve area, *MPG* mean pressure gradient, *MR* mitral regurgitation, *TR* tricuspid regurgitation, *PASP* pulmonary artery systolic pressure

### Long-term outcome of all patients and predictive factors

**Fig. 3:**
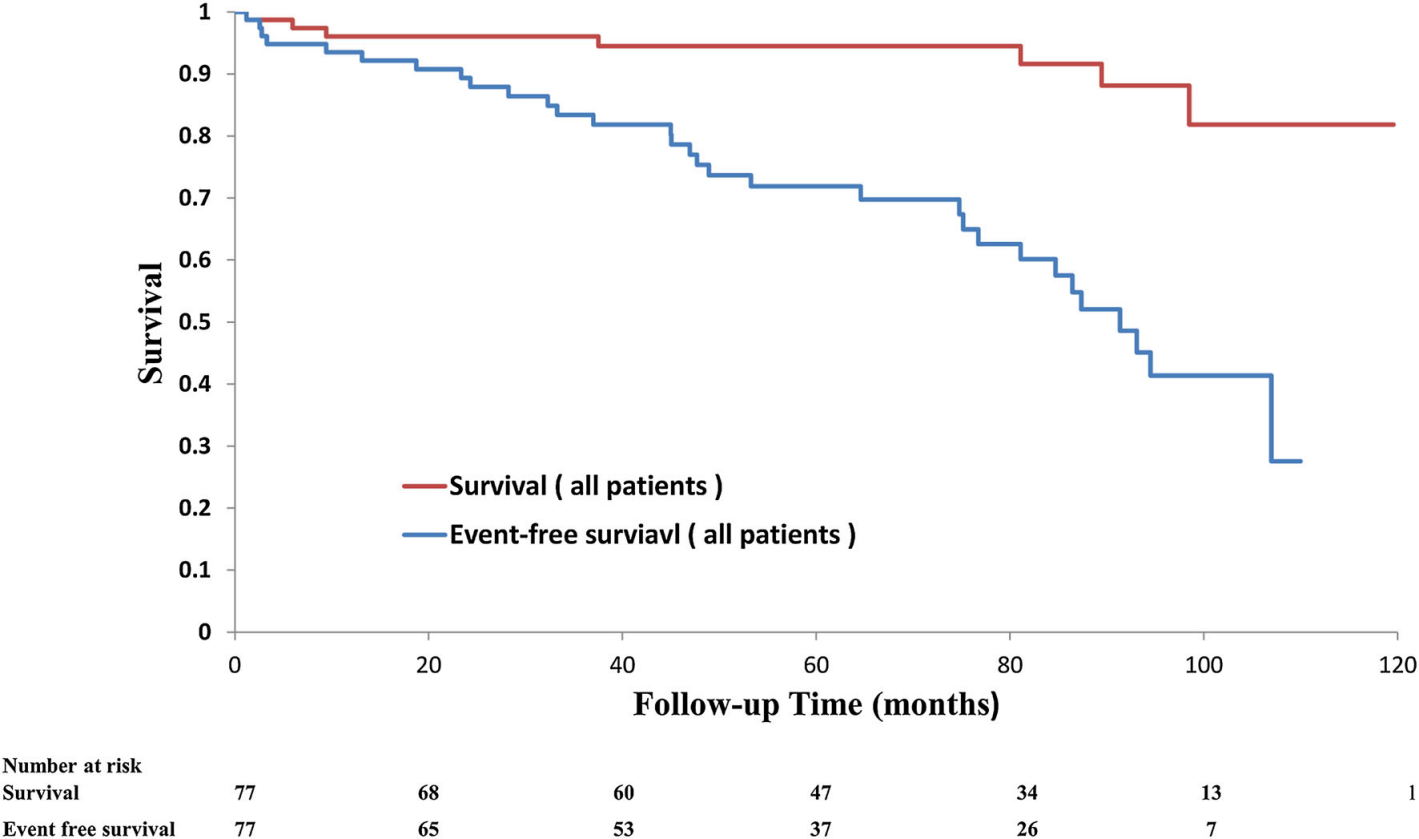
Kaplan–Meier curves of survival and event-free survival after successful PMV (*n* = 77). The clinical events were defined as all-cause mortality, hospitalization for HF, repeated PMV, MVR, and major CVA. *HF* heart failure, *PMV* percutaneous mitral valvuloplasty, *MVR* mitral valve replacement, *CVA* cerebral vascular accident

All patients completed clinical follow-up with a median follow-up of 73 months [interquartile range (IQR): 41–94 months]. The 1, 3, and 6-year rates of event-free survival of all patients were 93, 83, and 70 %, respectively. The 1, 3, and 6-year rates of survival of all patients were 96, 96, and 94 %, respectively (Fig. 3). The univariate predictors of clinical events at follow-up after successful PMV are detailed in Table 3. Age, sex, heart rhythm, and echocardiographic parameters were tested as a potential predictor of event-free survival using univariate Cox analysis and significant variables were categorized. None of the procedure-related variables was predictive. Multivariate analysis revealed that AF and PASP had an independent value.

**Table 3:** Univariate and multivariate Cox regression analysis predicting clinical events after successful PMV

Variables	Univariate			Multivariate		
	HR	95 % CI	*p* value	HR	95 % CI	*p* value
Age (≥62 years)^a^	2.11	1.03–4.59	0.041	1.81	0.87–3.96	0.109
Women	0.77	0.37–1.73	0.514	–		
NYHA class (III–IV)	1.34	0.58–2.90	0.489	–		
Atrial fibrillation	3.33	1.29–11.27	0.010	2.73	1.04–9.36	0.039
LAD (≥52 mm)^a^	1.50	0.73–3.07	0.267	–		
Pre-procedural MVA (≤1.1 cm^2^)^a^	0.84	0.39–1.73	0.647	–		
Post-procedural MVA (≤1.6 cm^2^)^a^	1.09	0.53–2.22	0.815	–		
PASP (≥39 mmHg)^a^	3.05	1.42–6.42	0.004	2.57	1.18–5.47	0.017
Echocardiographic score (≥8 points)	3.21	0.50–11.44	0.181			

*PMV* percutaneous mitral valvuloplasty, *NYHA class* New York Heart Association Functional Class, *LAD* left atrial diameter, *MVA* mitral valve area, *PASP* pulmonary artery systolic pressure, *HR* hazard ratio, *CI* confidence interval

^a^Continuous variables were categorized into two subgroups according to median values

### Comparison of long-term outcomes between AF and SR

**Fig. 4:**
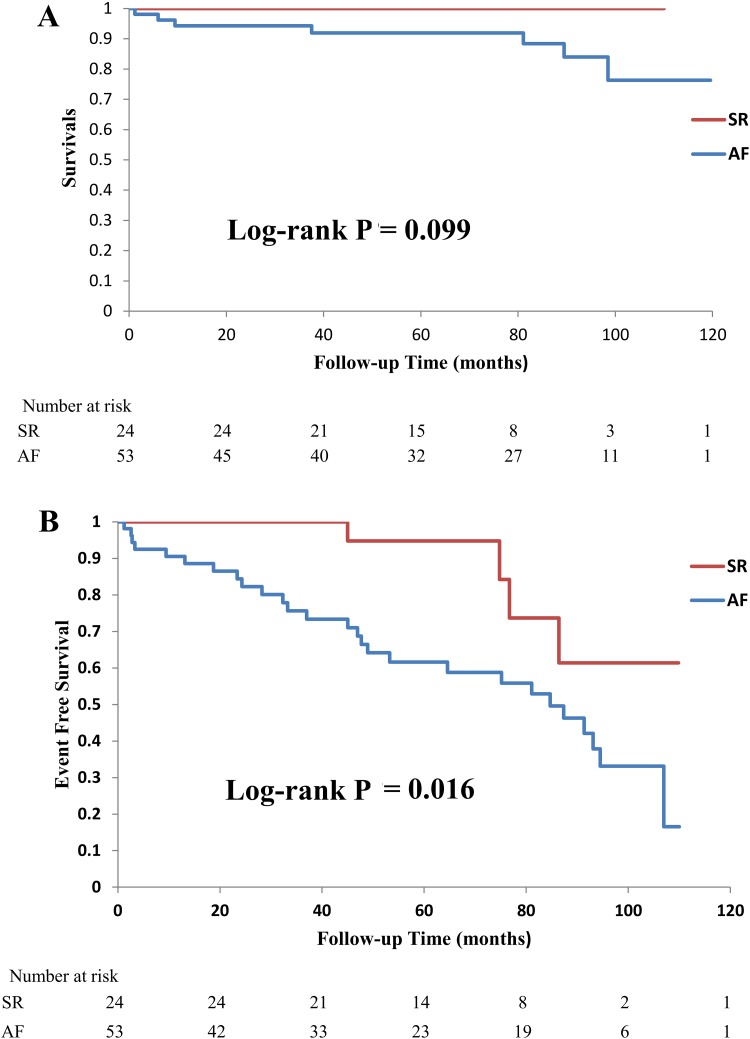
Kaplan–Meier survival curves free from **a** all-cause mortality, **b** clinical events in SR (*n* = 24) and AF (*n* = 53) groups after successful PMV. *PMV* percutaneous mitral valvuloplasty, *SR* sinus rhythm, *AF* atrial fibrillation

During the median follow-up period, there were no all-cause mortalities in the SR group, whereas 7 patients died in the AF group due to the following reasons: HF (2), acute myocardial infarction (1), bacterial pneumonia (1), gastrointestinal bleeding (1), liver cirrhosis (1), and sudden death (1). Survival rates in the AF group were 94, 94, and 92 % at 1, 3, and 6 years. AF group in comparison with SR group demonstrated a trend toward a higher survival rate (*p* = 0.099) that was not statistically significant. These results are shown in the Kaplan–Meier plots in Fig. 4a. The 1-, 3-, and 6-year rates of event-free survival between the two groups were (SR vs. AF) 100 vs. 91 %, 100 vs. 76 %, and 95 vs. 59 %, respectively. The event rate was significantly higher in the AF group compared with that of the SR group (*p* = 0.016) (Fig. 4b). The clinical events that occurred in both the groups during the median follow-up period are listed in Table 4. There were more mortalities and more interventions in the AF group, although the difference was not statistically significant, except for mitral valve surgery. In the present study, we added major CVA as a clinical event. The estimated actuarial 6-year rates of CVA-free survival were 95 % in the SR group and 89 % in the AF group. The incidence of major CVA tended to be more frequent in the AF group, although the difference was not statistically significant (*p* = 0.51). In addition, 9 out of 24 patients in the SR group newly developed chronic or persistent AF within the follow-up period, but there was no significant difference in both the survival and event-free survival rates between the new-onset AF group (*n* = 9) and the rest of the SR group (*n* = 15) (*p* = 1.0; *p* = 0.25, respectively).

**Table 4:** Clinical events at the median follow-up

Clinical events	Total (*n* = 77)	SR (*n* = 24)	AF (*n* = 53)	*p* value
All-cause mortality	7 (9 %)	0 (0 %)	7 (13 %)	0.06
HF for admission	9 (12 %)	1 (4 %)	8 (15 %)	0.16
Mitral valve replacement	14 (18 %)	1 (4 %)	13 (25 %)	0.03
Repeated PMV	5 (6 %)	1 (4 %)	4 (8 %)	0.50
Major CVA	7 (9 %)	1 (4 %)	6 (11 %)	0.29

Data are *n* (%)

*SR* sinus rhythm, *AF* atrial fibrillation, *HF* heart failure, *PMV* percutaneous mitral valvuloplasty, *CVA* cerebral vascular accident

## Discussion

AF adversely affects immediate and long-term results of PMV for significant MS [15, 16]. A major feature of the present study is that patients with a history of PMV and open mitral commissurotomy were included and patients with unfavorable mitral valve morphology for PMV (echocardiographic score ≥10) were excluded. Adverse clinical events included HF requiring hospitalization, major CVA, mortality, and repeated PMV and MVR. In such a clinical setting, our present study provides beneficial information related to daily clinical practice, because between the two groups with similar baseline MS, patients in preprocedural AF had similar immediate optimal results compared with those in SR. However, AF was associated with shorter long-term and event-free survival.

During clinical follow-up after PMV, the actuarial 6-year rates of survival without any-cause mortality, any subsequent intervention on the mitral valve, and any CVA were 70 % after successful PMV. Interestingly, the survival rate of all patients during the follow-up period was >90 %, which seems acceptable relative to those reported by others [17, 18]. However, approximately 40 % of all patients developed some clinical event within 7 years after successful PMV (Fig. 3). In our population, clinical outcome was strongly related to the baseline characteristics of the patients, particularly preprocedural AF and PASP, rather than post-procedural immediate results or baseline severity of MS. These data suggest that development of a clinical event was not always related to progression of severity of MS.

The incidence of AF in patients with MS is approximately 40 % [19]. AF associated with rheumatic heart disease differs in pathophysiology from that of AF in non-rheumatic disease. Age is a predominant factor in the development of AF, and left atrial enlargement may be the result, rather than the cause, of AF. The severity of MS is not invariably related to the incidence of AF [20], which is consistent with our finding that there was no difference between the two groups in MVA and MPG at baseline and after the procedure. Fawzy et al. reported that PMV in patients with AF achieved an inferior immediate result that was reflected by a lower success rate and a smaller post-PMV MVA [15, 16]. These different results may be explained by our selection of patients with valve morphology suitable for PMV. In the studies cited, echocardiographic scores of patients with AF were worse compared with their population selected by Fawzy et al. at baseline. Further, it is still unclear that AF has an adverse effect on the technical success of PMV. Therefore, 7 patients with major complications after PMV were excluded from our population.

The AF group showed higher preprocedural NYHA class, larger left atrial size, and higher echocardiographic score compared with those of the SR group. These findings indicate that left atrial remodeling progressed in the AF group compared with the SR group, even in those with similar severities of MS. Other study supports this idea, demonstrating that left atrial diameter independently predicted incidents of cardiovascular events [21]. According to the report investigated by Bouleti et al. [22], significant interaction between rhythm and NYHA class was observed only in patients with AF. Such patients with “advanced” MS may be significantly associated with a higher NYHA class and higher incidence of clinical events. Peter et al. [20] showed that hemodynamic measurements are not significantly different between patients with SR and those with chronic AF, with the single exception of lower cardiac outputs of patients with established AF. A low output state may be related to worse symptoms and functional capacity in patients with MS accompanied by AF compared with patients with SR.

Here, we demonstrate that the survival of patients with preprocedural AF was shorter compared with those with preprocedural SR. No patient with SR died after successful PMV during our study. These results are somewhat better compared with those of a previous report with survival rates of 89 % for the SR group and 68 % for the AF group with a mean follow-up of 5 years [16]. This difference is may be explained by the suitability of our population for PMV (echocardiographic score ≤9), as only those patients who achieved primary success were enrolled in our study. In our institute, almost all patients with echocardiographically unfavorable mitral valve for PMV and major complications after PMV were referred to surgical options. The incidences of MVR and repeated PMV were higher in the AF group compared with those of the SR group. Therefore, these data suggest that the recurrence rate of MS was higher in the AF group, even if the immediate results were similar to those of the SR group.

Advanced age, baseline NYHA class IV, high echocardiographic score (≥9), history of surgical commissurotomy, and mitral valve calcification are associated with improved suboptimal immediate outcomes and fewer long-term clinical events after PMV [23]. However, these parameters were not statistically significant in our study. The strong association between age and AF in MS suggests that the structural changes in the atrial myocardium that predispose to AF are time-dependent. Maatouk et al. showed that AF was an independent predictor of overall mortality and combined events. Their study population is worth noting, because patients with AF were much older compared with those with SR (44 ± 12 vs. 30 ± 13 years) [18]. On the contrary, Leon et al. reached at a conclusion that the presence of AF alone does not influence the long-term outcome, although there were significant differences at baseline in both groups (62 ± 12 years for AF vs. 48 ± 14 years for SR) [16]. Notably, the mean age of our patients in both groups was 62 years, and 67 % still had AF at baseline. A simple comparison between these studies is obviously not possible due to differences in patient characteristics. However, we speculate that AF may have a more adverse effect on long-term outcome of older patients. Further studies are required to clarify the effect of PMV on long-term outcomes according to age.

Early PMV has not been shown to be universally successful in preventing AF. In the present study, nearly 40 % of the SR group presented with AF at their final visit after successful PMV, which may be explained by the complex nature of AF in MS. Therefore, the optimal timing of PMV remains controversial for asymptomatic patients with significant MS and SR. Nonetheless, our data indicate the possibility that performing PMV before the onset of AF provides long-term clinical benefit through a complex mechanism other than maintaining SR. Moreover, patients with SR are associated with fewer symptoms compared with those with AF, as demonstrated in our data.

However, early intervention should be considered for SR patients with moderate MS and minimal symptoms, instead of watchful waiting for worsening of symptoms or developing a new-onset AF. A study that supports these recommendations found that in asymptomatic patients with moderate MS and favorable valve morphology, the estimated actuarial 11-year event-free survival rate was 89 % in the PMV group and 69 % in the conventional management group (*p* < 0.001), suggesting that the clinical benefits of early PMV may outweigh the risk associated with early intervention [24]. Prospective randomized trials are required to confirm the efficacy of early PMV.

### Study limitations

There were a few limitations in this study. First, this is a retrospective study of the records of a small number of patients from a single institution. Similar to other developed countries, the number of patients in Japan with MS requiring PMV is decreasing, along with the decline of the incidence of rheumatic heart disease [25]. In contrast, there are an increasing number of patients who require repeat treatment after undergoing PMV or surgical mitral commissurotomy. Therefore, we did not exclude such patients from our study. Second, we demonstrated that 59 % of the SR group had AF at the final visit after successful PMV. Further, we did not conduct a systematic study of the differences between persistent SR patients and those with new-onset post-procedural patients with AF, although the latter have some associations with prognosis or clinical events. However, defining patients with consistent SR is not trivial, because they can present with subclinical, transient AF. Further prospective randomized studies are required to confirm our results.

## Conclusions

PMV is a useful palliative procedure with excellent long-term results. Patients with AF were significantly associated with worse symptoms and higher event rates after undergoing successful PMV compared with those with SR. Patients with AF should be followed carefully for an extended time after successful PMV. Moreover, appropriate management of AF in patients undergoing PMV for MS may represent an important next step to alleviate symptoms or improve clinical outcomes.
